# Kinesiology, Physical Activity, Physical Education, and Sports through an Equity/Equality, Diversity, and Inclusion (EDI) Lens: A Scoping Review

**DOI:** 10.3390/sports10040055

**Published:** 2022-04-07

**Authors:** Khushi Arora, Gregor Wolbring

**Affiliations:** 1 Faculty of Kinesiology, University of Calgary, 2500 University Drive NW, Calgary, AB T2N1N4, Canada; khushi.arora@ucalgary.ca; 2 Community Rehabilitation and Disability Studies, Department of Community Health Sciences, Cumming School of Medicine, University of Calgary, 3330 Hospital Drive NW, Calgary, AB T2N4N1, Canada

**Keywords:** equity, diversity, and inclusion, equity, equality, diversity, inclusion, sport, sports, kinesiology, physical activity, physical education

## Abstract

Background: Equity, equality, diversity, and inclusion are terms covered in the academic literature focusing on sports, kinesiology, physical education, and physical activity, including in conjunction with marginalized groups. Universities in many countries use various EDI policy frameworks and work under the EDI headers “equality, diversity and inclusion”, “equity, diversity and inclusion”, “diversity, equity and inclusion”, and similar phrases (all referred to as EDI) to rectify problems students, non-academic staff, and academic staff from marginalized groups, such as women, Indigenous peoples, visible/racialized minorities, disabled people, and Lesbian, Gay, Bisexual, Transgender, Queer or Questioning, and Two-Spirit (LGBTQ2S+) experience. Which EDI data, if any, are generated influences EDI efforts in universities (research, education, and general workplace climate) of all programs. Method: Our study used a scoping review approach and employed SCOPUS and the 70 databases of EBSCO-Host, which includes SportDiscus, as sources aimed to analyze the extent (and how) the academic literature focusing on sports, kinesiology, physical education, and physical activity engages with EDI. Results: We found only 18 relevant sources and a low to no coverage of marginalized groups linked to EDI, namely racialized minorities (12), women (6), LGBTQ2S+ (5), disabled people (2), and Indigenous peoples (0). Conclusions: Our findings suggest a gap in the academic inquiry and huge opportunities.

## 1. Introduction

Many different EDI-related phrases, such as “equity, diversity and inclusion”, “diversity, equity and inclusion”, and others [1,2,3,4,5,6,7,8,9,10,11,12,13,14,15,16,17,18,19,20,21,22], and EDI frameworks, such as Athena SWAN (Scientific Women’s Academic Network) [23], Australia (Science in Australia Gender Equity, SAGE-Athena SWAN) [24], the USA (See change with STEMM Equity Achievement, SEA-Change [25] and NSF ADVANCE [26]), and Canada (DIMENSIONS: Equity, diversity, and inclusion) [27]) are used to engage with equity/equality, diversity, and inclusion problems students, academic staff, and non-academic staff of marginalized group, such as women, Indigenous peoples, visible/racialized minorities, disabled people, and Lesbian, Gay, Bisexual, Transgender, Queer or Questioning, and Two-Spirit (LGBTQ2S+), experiences in higher education, including in programs focusing on sports, kinesiology, physical education, and physical activity. EDI phrases are also employed by groups focusing on sports, kinesiology, physical education, and physical activity outside universities settings [28,29,30,31,32,33]. What EDI data, if any, is generated within the academic literature focusing on sports, kinesiology, physical education, and physical activity can influence the implementation and direction of EDI focusing on sports, kinesiology, physical education, and physical activity in universities (research, education, and general workplace climate) and outside. Therefore, we used a scoping review approach to analyze to what extent (and how) the academic literature that focuses on sports, kinesiology, physical education, and physical activity engages with EDI. Our two main research questions were: (1) which EDI frameworks and phrases are present in the academic literature focusing on sports, kinesiology, physical education, and physical activity engages? (2) What themes, and which EDI marginalized groups, are present in the EDI coverage in the sports, kinesiology, physical education, and physical activity focused academic literature? We discuss our findings through the lens of sports-, kinesiology-, physical education-, and physical activity-focused academic literature mentioning individual EDI terms. We also use literature around EDI policy frameworks and concept of ableism as lenses.

### 1.1. The Topic of Equity/Equality, Diversity, and Inclusion (EDI)

Many different EDI-related phrases have been generated in recent years, such as equity, diversity, and inclusion [1]; equality, diversity, and inclusion [1]; diversity, equity, and inclusion [1]; belonging, dignity, and justice [2,3]; diversity, equity, inclusion, and belonging [4,5,6]; employment equity [7]; equity, diversity, dignity, and inclusion [8]; equity, diversity, inclusion, and accessibility [9,10,11,12]; justice, equity, diversity, and inclusion [13,14,15,16,17,18]; inclusion, diversity, equity, and accessibility [9,11]; inclusion, diversity, equity, and accountability [19,20,21]; and equity, diversity, inclusion, and decolonization [22]. Furthermore, many EDI frameworks have been employed with the first being the 2005 Athena SWAN (Scientific Women’s Academic Network) [23] and others that followed, such as Australia (Science in Australia Gender Equity, SAGE-Athena SWAN) [24], the USA (See change with STEMM Equity Achievement, SEA-Change [25] and NSF ADVANCE [26]), and Canada (DIMENSIONS: Equity, diversity and inclusion) [27].

Work performed under these EDI frameworks and EDI phrases are envisioned to lead to systemic positive change for students, academic staff, and non-academic staff in universities as a workplace, in general, but also in the research and education reality [25]. Although the EDI focus is often on STEM (science, technology, engineering, mathematics) [25,26] and EDI started with a focus on gender equality [23,24,26], the EDI focus, by now, encompasses all areas of universities and various marginalized groups, such as women, Indigenous peoples, visible/racialized minorities, disabled people, and LGBTQ2S+ [27,34,35,36]. To quote from the Canadian EDI framework:

“Dimensions: equity, diversity and inclusion Canada invites you to take part in a post-secondary transformation to increase equity, diversity and inclusion (EDI) and help drive deeper cultural change within the research ecosystem” [35]. “The Dimensions program addresses obstacles faced by, but not limited to, women, Indigenous Peoples, persons with disabilities, members of visible minorities/racialized groups, and members of LGBTQ2+ communities” [35].

However, many problems have been identified, in relation to EDI implementations [1].

### 1.2. The Individual Concepts of Equity, Equality, Diversity, and Inclusion in Sport

Studies focusing on sports engage with equity and equality, in the context of the EDI groups of gender [37,38,39,40,41,42], race [43,44,45,46,47], and LGBTQ2S+ [48,49,50,51], including through an intersectionality lens [52] of these three identities [53]. Coverage exists for the term’s diversity and inclusion [54,55,56,57,58,59,60,61] and Indigenous peoples [62,63,64,65,66,67,68]. Studies noted that women especially in third world countries continue to experience a lack of equity in sport [69], that women’s sports are underrepresented in media coverage and that women are underrepresented in sports careers such as sports journalism, sports media, and sport leadership positions [70,71,72]. The literature covers race equity in sports, especially of athletes of color, in many ways, such as highlighting the excessive number of penalties [47] or pressuring of black students into athletics [43]. It is argued that a lack of diversity in sports is concerning because sport facilitates group cohesion; therefore, underrepresenting certain groups within sports can lead to further segregation of those groups [73]. There is an emphasis on the importance of ethnic diversity within sports, specifically youth sports, as a team sport provides an environment for children to come together from all different backgrounds and engage towards a common goal [74]. These experiences are beneficial, as they play a role in the identity formation of children as they age [75]. Discussions of inclusion in sports usually refers to social inclusion and emphasizes that sports are a facilitator for social inclusion and community engagement [76,77]. These implications are significant for groups that experience a lack of inclusion [78,79], whereby the lack of support initiatives for females and, specifically, initiatives for gender equity in sports must be addressed [80,81,82].

### 1.3. The Individual Concepts of Equity, Equality, Diversity, and Inclusion in Kinesiology

Studies focusing on kinesiology engage with equity and equality, in the context of the EDI groups of gender [83,84,85,86], race [87,88], and LGBTQ2S+ [89], including through an intersectionality lens [90,91] of these three identities [92,93]. It is suggested to perform equity audits [94]. Coverage exists for the terms diversity and inclusion [95], as well as for Indigenous peoples [91]. It is emphasized that “as our nation and society becomes more racially and ethnically diverse”, this diversity also translates to the “student demographic on campus”, meaning that the student body is becoming more diverse” [96] (p. 66). Therefore, it is important that the content of kinesiology education teaches reflects that diversity and addresses all kinds of people. However, it is argued that kinesiology education is biased towards whiteness [88]. Furthermore, it is highlighted that, even though students are becoming more diverse on campus, that diversity does not necessarily reflect the students in kinesiology programs [97]. It is recommended that kinesiology programs, both graduate and undergraduate, should make an effort to recruit more diverse students [97]. This need for diversity is addressed through the need for more ethnically diverse students, as well as more women in the faculty [98]. It is argued that if kinesiology programs include content on reducing inequalities and social justice, that will then help to “address societal problems within our communities” [92] (p. 271).

### 1.4. The Individual Concepts of Equity, Equality, Diversity, and Inclusion in Physical Education

Studies focusing on physical education engage with equity and equality, in the context of the EDI groups of gender [99,100], race [101], and LGBTQ2S+ [89,102,103], including through an intersectionality lens [104,105] of these three identities [106]. The same is true for the terms diversity and inclusion [107,108,109,110,111,112,113,114], as well as for Indigenous peoples [115,116,117]. It is argued that a focus on equity is needed in physical education curricula [118], because the educators learn about physical education through their university experiences [118]. It is argued that, in order to increase inclusivity within the classroom, the importance of that must be emphasized in training programs that physical education teachers must complete before they are eligible to work [119], and teachers have to be confident in generating inclusion in the classroom once they graduate [119], a confidence they are seen to lack [120]. It is also argued that physical education training programs must address issues regarding diversity and diversity attitudes [121].

### 1.5. The Individual Concepts of Equity, Equality, Diversity, and Inclusion in Physical Activity

Studies focusing on physical activity discuss equity and equality, in the context of the EDI groups of gender [42], race [122,123,124,125], and LGBTQ2S+ [126], including through an intersectionality lens [127,128,129,130] of these three identities [53]. The same is true for the terms diversity and inclusion [126], as well as for Indigenous peoples [62]. Regular physical activity is an important aspect in healthy living [131]; however, many “cities lack built environments that support physical activity” [132] (p. 1475). This is problematic because physical activity is a key indicator of health [132] and health equity [133]. Furthermore, socio-demographics influence whether one engages in physical activity [133]. For example, girls follow physical activity guidelines less than boys [133,134], and factors such as “race/ethnicity, household income, maternal education level, and perceived social status” [133] (p. 514) impact ones engagement in physical activities. With that, it is argued that a gender-neutral narrative, when addressing physical activity, is needed, and barriers originating with the social environment of a person have to be tackled [135].

### 1.6. The Individual Concepts of Equity, Equality, Diversity and Inclusion in Sports, Kinesiology, Physical Education, and Physical Activity: The Case of Disabled People

The *UN Convention on the Rights of Persons with Disabilities* flags access to recreation, leisure, and sport in their daily life, including schools [136], as a problem disabled people experience. Equity, equality, diversity, and inclusion, as individual terms, are discussed extensively, in the context of disabled people, within the academic literature focusing on sports, kinesiology, physical activity, and physical education [137,138,139,140,141,142]. Debates are ongoing as to the meaning of diversity [113], different approaches to diversity in physical education curricula [113], and benefits for disabled students being part of physical education classes [143]. National curricula for physical education are seen “as an important vehicle for social policy targeting the inclusion of disabled young people” [144] (p. 291); however, at the same time, many problems are reported to still exist in 2021 [144]. These problems are detrimental to disabled students [144], and it is noted that these problems generated barriers for physical education to benefit from the increased motivation of disabled pupils to partake in sports after the London 2012 Paralympics [144]. Many problems have been linked to how the physical education faculty engages with disabled people: faculty awareness of disability mandates, limited faculty training, lack of knowledge of federal disability mandates, negative reactions to disability disclosure, failure to accommodate, train and support faculty on academic adjustments, assistive technology, and teaching strategies, as well as the failure to ensure faculty comply with reasonable accommodations [145]. Problems have been not only reported in the context of physical education. It is argued that “ongoing advocacy, support, networking in raising awareness and promoting inclusion and equality in both mainstream and deaf/disability organizations are necessary to empower and increase participation and leadership roles for deaf/hard of hearing girls and women in sport” [146] (p. 71), and there is a need to increase formal coach education of parasport coaches [147]. According to the World Health Organization, “much work is needed to achieve equity in physical activity opportunities, access, and participation for people living with disability” [148] (p. 91). It is also argued that: there are many barriers to the inclusion of disabled people in kinesiology [149], non-disabled students have to be more exposed to disabled people and their social realities in kinesiology teaching [150], diversity and inclusion have to be clearly defined and cared for in kinesiology on all levels from students to faculty [151], more has to be done to generate more research that “counters deficit thinking from a social justice perspective” [152] (p. 225), and kinesiology researchers should “challenge dominant (majoritarian) discourses through critical interrogation of oppression (e.g., people of color, women, and individuals with disabilities) and privileges” [152] (p. 225).

#### The Issue of Ableism

Disabled activists and academics coined the term ableism in the United States and Britain during the 1960s and 1970s to flag the cultural reality of ability-based expectations, judgments, norms, and conflicts. Many worked, and work is ongoing on the disabling and enabling use of ability expectations and ableism [153,154,155,156,157,158,159,160,161,162,163,164,165,166,167,168,169,170,171,172,173,174,175,176], covering the relationship between ‘non-disabled people’ and ‘disabled people’, as well as humans–humans relationships, in general, humans-post/transhumans, humans-cyborg humans, humans-non sentient machines, humans-animals, and humans-nature relationships, linking ableism to many social theories and topics. Some ability concepts are: ability security (one is able to live a decent life with whatever set of abilities one has), ability identity security (to be able to be at ease with ones abilities), and ability inequity, an unjust or unfair (a) “distribution of access to and protection from abilities generated through human interventions” or (b) “judgment of abilities intrinsic to biological structures such as the human body” [156,177]. Ableism not only intersects with other forms of oppression, such as racism, sexism, ageism, and classism, but abilities are often used to justify such negative isms [153,154,168,178,179,180,181,182]. 

Ableism is also used to call out ability-based discriminations against disabled people within the kinesiology, sport, physical education, and physical education literature [91,149,151,183,184,185,186,187,188,189,190,191,192,193,194], including the intersectionality of ableism with other isms and prejudices [186]. As to kinesiology, one study found an able-bodied curriculum encouraging ableist attitudes among kinesiology students and an invisibility of disabled people [184]. Another study covering kinesiology education found prejudice, discrimination, microaggression, and deficit language against people with disabilities and structural ableism [185]. One article noted that many students might question the isms related to one group, such as gender, but do not, for example, question other isms they might internalize, such as racism or ableism [187].

As to physical education, instructional ableism and microaggressions is flagged as a problem [191], and it is argued that physical education should enable critical engagement with ableism, which would entail a “different ability curriculum, which destabilises notions of normativity and challenges the status quo regarding ability” [194] (p. 518) and understanding that “the diversity of humanity through a ‘differently-abled’ framework as well as to critique the construction of disability from a deficit perspective” [193] (p. 1). Ableism is flagged as a useful analytical perspective in sports pedagogy to reflect on its discriminatory and exclusionary potential [149]. It is argued that there are competing ability-related narratives, such as the “global trend for (normalized) physical self-optimization on the one hand, and the struggle to achieve acceptance of (bodily) diversity triggered by the societal claims for inclusion on the other hand” [149] (p. 152). It is argued that ableism, as a lens, highlights that “stigmatisation, exclusion and disability cannot be ascribed to the individual; these are instead first actively generated by the discursive body image” [149] (p. 158) and reflects that “traditionally, imperfection has had no place in the pedagogy of PE” [149] (p. 155), as well as that PE teachers “instrumentalize the body” [149] (p. 162) and “tend to focus on deficits when dealing with persons whose bodies deviate from the norm” [149] (p. 162), which is seen as “an obstacle for full and equal participation for all young people in PE at school” [149] (p. 162).

Regarding sports, it is argued that ableism impacts social practice within sports, and ableism in sports has to be addressed [188,195]. It is argued that experiences and discourses around sports mostly internalize and take ableism-based identities as a given [183,188,190], and ableism is one reason for why disability sports have largely been ignored, in the context of diversity management in sports [196]. It is argued that it is critical to understand “ableism and how ableistic ideology informs sporting theory, sport science and sport management” [197] (p. 5).

Many studies cover individual EDI terms that make up the various EDI phrases in the context of sports, kinesiology, physical education, and physical activity. However, to our knowledge, no study has, so far, analyzed whether the academic literature also engages with the existing EDI phrases and frameworks. That is important to know because EDI phrases and frameworks are linked to specific policy endeavors in the workplace, such as universities. How these policies are implemented impacts the research and education activities of and day-to-day operations in the fields of sports, kinesiology, physical education, and physical activity. Our study aims to fill the gap and investigate to what extent (and how) the academic literature focusing on sports, kinesiology, physical education, and physical activity engages with existing EDI phrases and frameworks. Given that specific marginalized groups are the focus of the activities performed under the EDI phrases and frameworks, our study also investigated which marginalized groups are mentioned.

## 2. Materials and Methods

### 2.1. Research Design

Scoping studies are useful in identifying the research that exists on a subject [198,199]. Our scoping study focused on the research that engages with EDI, in the context of sports, kinesiology, physical education, and physical activity. Our study was guided by the very policy premises of EDI [1].

Our study employed a modified version of a scoping review outlined by Arksey and O’Malley [200], as performed in another study [201]. Our research questions were: (1) Which EDI frameworks and phrases are present in the academic literature focusing on sports, kinesiology, physical education, and physical activity engages? (2) What themes and which EDI marginalized groups are present in the EDI coverage in the sports, kinesiology, physical education, and physical activity focused academic literature?

### 2.2. Data Sources and Data Collection

We searched, on 22–26 May 2021 (14 December 2021, for strategy 2), the 70 databases accessible through EBSCO-Host, which includes CINAHL, SportDiscus, and Scopus (which incorporates the full Medline database collection) for English language data with no time restrictions, accessing journals that cover relevant content to our research questions. We searched for scholarly peer-reviewed journal articles in EBSCO-Host, and we searched for reviews, peer-reviewed articles, conference papers, and editorials in Scopus. We performed the following search strategies (Table 1).

### 2.3. Data Analysis

To answer the research questions, we first obtained hit counts for our search term combinations (Table 1), employing a descriptive quantitative analysis approach [202,203]. We obtained the abstracts, as part of downloading the citations obtained through Scopus and EBSCO-Host, using the Endnote 9 software. We removed duplicates within the Endnote 9 software and exported the remaining abstracts, as a Word file, from the Endnote 9 software. We then uploaded the Word file with the abstracts into the qualitative analysis software ATLAS.Ti 9™ for directed qualitative content analysis [202,203,204,205] of the data focusing on the research questions, meaning the abstracts had to cover EDI as a policy framework and not just engage with individual EDI terms. Both authors coded the abstracts to see which fit the inclusion criterium, and the full text articles of these abstracts were downloaded and uploaded into ATLAS.Ti 9™. We used a directed content analysis to add knowledge about the phenomenon of EDI and areas of kinesiology, physical education, physical activity, and sports that benefit from further description [202]. As to the coding procedure, beyond having judged the relevance of the abstracts for downloading the full texts, we followed a procedure we used before [201], as outlined by others [202,205,206].

### 2.4. Trustworthiness Measure

As to trustworthiness measures [207,208,209], the few differences in the analysis of the qualitative data were discussed and resolved between the authors (peer debriefing) [209]. Confirmability was achieved by using the audit trail, employing the memo and coding functions within ATLAS.Ti 9™ software. As for transferability, we provided all the information needed, so that others can decide whether to apply our study design to other sources or change design aspects, such as different keywords.

## 3. Results

**Figure 1:**
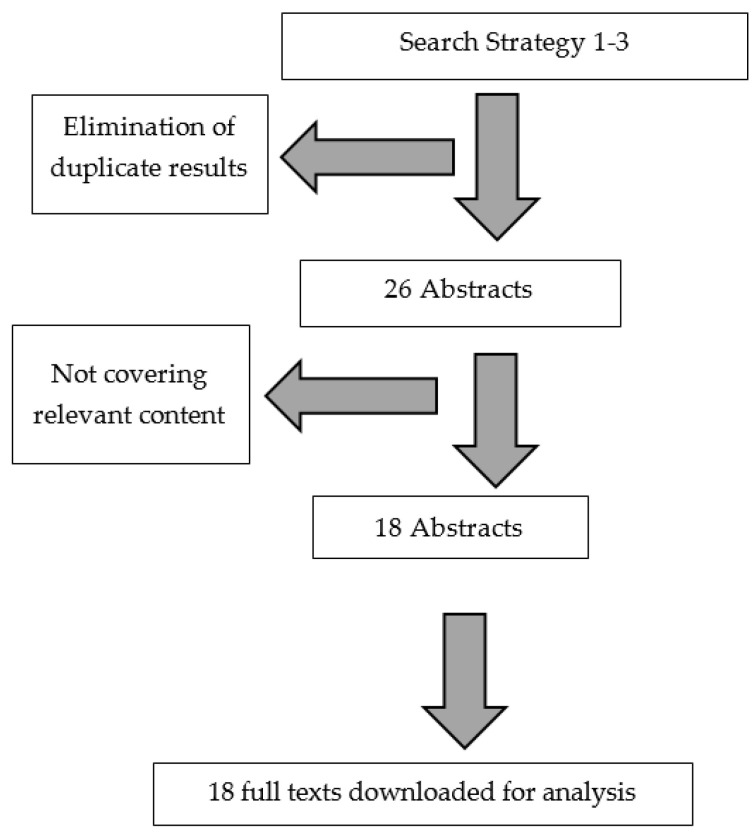
Flow chart of the selection of academic full texts for qualitative analysis.

The search strategies generated 26 abstracts, of which 18 were deemed to have relevant content. The full texts of the 18 relevant abstracts were downloaded and thematically analyzed (Figure 1). 

We present the themes in the downloaded full text articles in three sections:

(a) The first one being academic/educational setting, but not university, which was classified as anything that is related to academics (but not specifically to a university) setting; for example, research conferences that are open to all fields of studies and careers, K to 12 education, and other academic organizations.
(b) Non-academic settings, which primarily looked at sport facilities and organizations, recreational facilities and organizations, and general physical activity.
(c) University setting, consisting of discussions around different university institutions and, specifically, different areas of the faculty of kinesiology.

We separated the findings further by the following four areas: physical education, sport, physical activity, and kinesiology.

Furthermore, we separate the findings into the four common themes we found: (a) EDI recommendation/EDI needs, (b) EDI curriculum and teacher/educator/mentor role in EDI, (c) EDI literacy/EDI narrative, and, lastly, (d) EDI study results.

Finally, we separated the results according to which EDI group they looked at. If a source did not specifically discuss equity deserving groups, it was labelled as “no group”.

In each of the results sections, we first provide the frequency counts for the presence of themes we found. We only list where there was at least one hit for the theme. The themes that had 0 results are not listed in the tables.

### 3.1. Academic/Educational Setting

Within the academic/educational setting theme, the following themes returned no results and, as such, are neither listed in Table 2 and Table 3 nor reflected in the sub-headers:

- 0 results on physical education, in terms of EDI study results;
- 0 results on sport, in terms of EDI curriculum and educators and mentor’s role in EDI;
- 0 results on physical activity, in terms of EDI recommendations/EDI needs;
- 0 results on physical activity, in terms of EDI curriculum and educators and mentor’s role in EDI;
- 0 results on physical activity, in terms of EDI literacy/EDI narrative;
- 0 results on physical activity, in terms of EDI study result;
- 0 results on kinesiology, in terms of ALL the EDI-related themes.

#### 3.1.1. Academic Setting and Physical Education

##### EDI Recommendation/EDI Needs

There were twelve findings, in a total of five sources, that discussed EDI recommendations/EDI needs, in the context of physical education in an academic setting. Out of the twelve findings, one of them discussed persons with disabilities, three of them discussed ethnic groups, and eight of them did not cover any specific EDI group.

The EDI recommendation related to persons with disability(s) in physical education settings was that regular contact with non-disabled children in physical education classes aids in the inclusion of those with disabilities in society [210]. The EDI recommendation related to ethnic groups discussed that, if students limit their interactions with the same or similar cultural backgrounds in the physical education class, it is then reflected in the student’s behavior and interactions in society [210]. This statement essentially emphasizes that it is important that students do not limit their interactions with other students of different cultural backgrounds and highlights that, if this type of behavior is happening within the classroom, it is very likely that it will also occur outside of the classroom [210]. Another recommendation that looked at including ethnic groups was that, in order to make education more accessible, we should look at multilingual or cultural resources that can engage more communities and, as a result, have more participants engaging in learning [211]. In addition, another recommendation for the inclusion of ethnic groups discussed the importance of culturally sensitive and inclusive pedagogy in the classroom [210]. Other recommendations that discussed physical education in an academic setting did not address a specific group covered by the EDI framework but discussed other EDI recommendations and the need for EDI in these settings. Firstly, the need for EDI was addressed through the importance of inclusive education. Specifically, it is noted that inclusive education means that all learners, no matter who, should have access to mainstream education, and inclusive education should benefit all learners [211]. A recommendation for inclusive learning stated that there are many resources available online for learning that can aid education to be more inclusive [211]. Recommendations for EDI and the need for EDI was also looked at, specifically in the physical education classroom and curriculum. For example, a recommendation emphasized the importance of establishing an inclusive classroom environment, where all students feel like they are included and belong in the classroom [210]. Another major recommendation was the need for educational curricula to cover EDI topics, so students could be educated on the importance of diversity, equity, and inclusion [212]. To keep those creating curricula accountable, a recommendation was put forward that, if curricula fail to implement EDI frameworks, there should be consequences for such actions [213]. The need for EDI was also addressed outside of the classroom, specifically in certain research journals [214]. This study looked at one journal, i.e., *The Recreational Sports Journal*, and found that EDI was not discussed in a meaningful way during the time period of their study; it is very important to address this void and publish more papers that cover EDI topics [214]. It was recommended in this paper that the editor-in-chief and editorial board of the journal should generate a call for papers that are focused on EDI issues, as well as designate research funding to the topic of EDI [214].

##### EDI Curriculum and Teacher/Educators/Mentors Role in EDI

There were nine findings, in a total of three sources, that discussed EDI curricula and the teachers/educator/mentor role in EDI, in the context of physical education in an academic setting. Out of the nine findings, one of them discussed ethnic groups and the other eight covered no specific EDI group. In terms of ethnic groups, it was discussed that taking the initiative to build EDI curricula brings the opportunity to include more culturally responsive and cultural enrichment pedagogy [211]. The following findings did not discuss a specific EDI group but did cover the idea of an EDI curriculum and educators and mentors role in EDI. It was discussed that educators and mentors must make sure that everyone feels welcome, supported, and valued in their space, so students can achieve their goals and grow their talents [211]. Understanding the value that physical education has, in terms of building relationships across different groups of people, was addressed [210]. The idea that educators must reflect on their teaching styles and activities within the physical education classroom was highlighted [215]; in order to do so, it was said that this requires a deep understanding of the barriers and societal issues that minority groups face [210]. There was further emphasis on the fact that it is important that educators also teach for social cohesion and, when doing so, they must be careful to be aware of dominant versus minority groups and teach accordingly [210]. Overall, the idea that teachers must be aware of the impact that implementing EDI-based curricula can have for the children outside of the classroom, as well as the role they play in achieving positive outcomes, has been noted as crucial.

#### 3.1.2. Academic Setting and Sports

##### EDI Recommendation/EDI Needs

There were three findings, out of a total of one source, that discussed EDI recommendation/EDI needs, in the context of sports in an academic setting. Out of those three findings, none of them covered a specific EDI group. The source was a statement that was put out by the *Journal of Sport Rehabilitation* for their commitment to diversity, equity, and inclusion [216]. In the statement, they addressed the importance of diversity, equity, and inclusion in research and provided some future recommendations and goals for their own journal [216]. They started off by openly embracing the concept of EDI and said that they want to maintain a culture that embraces EDI within their journal [216]. They further went on to say that they want to work actively to promote change through purposeful EDI initiatives, as well as publish more diverse research that can be translatable to a more patient diverse population [216].

##### EDI Literacy/EDI Narrative

The following source discusses reflections that occurred at EDI conferences [217]. This source did not specifically cover physical education, physical activity, sports, or kinesiology, but they did take the reflections mentioned in the conferences and mentioned them in the context of sports; therefore, we categorized these findings in this section. There are four findings in the one source that discussed EDI literacy and narratives in an academic setting, specifically research conferences. None of these findings discussed a specific EDI group. This source first started off by highlighting that one of the barriers to EDI literacy is connected to the fact that the EDI research that is broadcasted and discussed during conferences is not easily accessible for the vast majority of people who do not get invited to conferences; therefore, it is important that we have literature for people to learn about EDI through credible sources [217]. There was further discussion on the idea that statistics cannot represent the actual stories that are behind individual answers, and its stories can show us the intersectionality in a clear way [217]. In addition, it was said, in relation to EDI literacy, that positive stories can often deflect from the negative stories that make people uncomfortable; therefore, this can affect the EDI narrative [217]. With that in mind, it was said that it is important that we provide appreciative inquiry and not critical inquiry when we are analyzing negative stories relating to EDI [217]. 

##### EDI Study Result

There were seven findings, out of a total of two sources, that discussed EDI study results, in the context of sports in an academic setting. One of the sources covered all EDI groups, whereas the other source did not cover any EDI groups. The first source was a content analysis of equity, diversity, and inclusion in the *Recreational Sports Journal* [214], performed for the years of 2005 to 2019. The study results revealed that reporting participants gender was the only variable that showed improvement from the pre-to-post-EDI commission era [214]. In this content analysis, it was also highlighted that the most to least discussed issues in EDI research was in order from gender, race and ethnicity, social class, nationality issue, sexual orientation issue, disability issue, and non-traditional student issues [214]. It is important to take note that, out of the EDI groups we are looking at, the content analysis clearly highlighted that disability issues were the least discussed in EDI research. It was highlighted in another statement that gender was the most addressed cultural diversity issue, and there is little attention to topics, such as disability, nationality, race and ethnicity, sexual orientation, or social class [214]. This content analysis also revealed that, out of the 200 *Recreational Sports Journal* articles that were analyzed, 18% addressed at least one equity diversity and/or inclusion issues [214]. Furthermore, it was also revealed that EDI issues were addressed in less than 20% of the articles in the journal [214]. The second source looked at the development of a model of diversity, equity, and inclusion for support volunteers [218]. In this source, it was revealed that sports volunteers experience a lack of inclusion on many levels and microaggressions [218]. It was specifically discussed that there is limited research that critically examines the experience of volunteers that belong to traditionally underrepresented populations [218].

### 3.2. Non-Academic Setting

Within the non-academic setting theme, the following returned no results and, as such, are neither listed in Table 4 and Table 5 nor reflected in the sub-headers:

- 0 results on physical education, in terms of all the EDI-related themes;
- 0 results on sports, in terms of EDI curriculum and educator/mentor’s role in EDI;
- 0 results on sports, in terms of EDI literacy/EDI narrative;
- 0 results on physical activity, in terms of EDI curriculum and educators/mentor’s role in EDI;
- 0 results on physical activity, in terms of EDI literacy/EDI narrative;
- 0 results on physical activity, in terms of EDI study results;
- 0 results on kinesiology, in terms of ALL the EDI-related themes.

#### 3.2.1. Non-Academic Setting and Physical Activity

##### EDI Recommendation/EDI Needs

There was one finding, through one source, that discussed EDI recommendations/EDI needs in the context of physical activity in a non-academic setting. This EDI recommendation/EDI need did not cover any specific EDI group. It was discussed that there is an “urgent need” to address EDI within intramural and recreational sports [214]. This recommendation and expression for the need of EDI also covers the topic of sports; however, we also added it in the physical activity section because intramural and recreational sports are commonly a form of leisure time physical activity and usually do not include those who play sports professionally.

#### 3.2.2. Non-Academic Setting and Sports

##### EDI Recommendation/EDI Needs

There were five findings, in a total of four sources, that discussed EDI recommendations/EDI needs in the context of sport in a non-academic setting. Out of those five findings, one discussed women and ethnic groups, one discussed ethnic groups, and two discussed no EDI groups. The EDI recommendation and need to promote racial EDI within black women’s football in Brazil were highlighted [219]. This recommendation emphasized the importance of EDI, in the context of women, as well as ethnic groups. The EDI recommendation that was specific to ethnic minorities was that, without intentional recognition and efforts towards addressing racial disparities, we will not see meaningful progress, in terms of the leadership efforts, for EDI and, in some cases, could even result in more damage, if we do not address racial disparities [213]. This paper was specific to college sports and sport leadership; however, the paper applied this statement generally and, therefore, is categorized under the non-academic section. In terms of general sports in a non-academic setting, it was said that there is an urgent need to start addressing EDI-related audiences, issues, and topics within the field of intramural and recreational sports [214]. Furthermore, it was recommended that national governing bodies should consider implementing mentorship programs, take steps to limit the influence of social connections on advancement decisions, reduce barriers to participation, and provide training to reduce the presence of microaggressions and unconscious bias within sport [218]. Lastly, the need for EDI was highlighted by saying that an EDI framework within sport organizations is necessary for success of that sport organization [218].

##### EDI Study Result

There were eight findings, out of a total of three sources, that presented EDI study results in the context of support in a non-academic setting. Out of the eight findings, four discussed LGBTQIA+ topics, two discussed women, one discussed ethnic groups, and one discussed both women and ethnic groups. One EDI study result discussing LGBTQIA+ was that athletes have a fear of disclosing their sexuality or sexual orientation, in fear of discrimination from the sports industry [220]. Because of this, players will not disclose their non-heterosexual orientation, in order to avoid homophobic discrimination from their fans, agents, the media, and, lastly, their employers [220]. Furthermore, there was discussion on the question as to whether the equity law, as well as the law on positive action, are enough to promote the equality of treatment and opportunity, when it comes to the employment of LGBTQIA+ elite sport professionals, specifically football players [220]. Lastly, it was said that the acknowledgement of the presence and prevalence of homophobia in football is a more recent phenomenon [220]. An EDI study result highlighted that female representation and recognition in sports have yet to catch up to that of their male counterparts [221]. Furthermore, there is limited coverage that is specific to women’s sports; when women are presented as a topic, they are faced with arbitrary issues, such as femininity and sexuality [221]. Specific to ethnic groups, a EDI study result highlighted that, even though many professional athletes are people of color, those who want to be employed as coaches and managers are still facing discrimination within sport [220]. One of the EDI study results that covered both ethnic groups and women was that there is white male advantage in sports [217].

### 3.3. University Setting

Within the university setting theme, the following returned no results and, as such, are neither listed in Table 6 and Table 7 nor reflected in the sub-headers:

- 0 results on physical education, in terms of ALL the EDI-related themes;
- 0 results on sports, in terms of EDI curriculum and educator/mentor’s role in EDI;
- 0 results on sports, in terms of EDI literacy/EDI narrative;
- 0 results on sports, in terms of EDI study result;
- 0 results on physical activity, in terms of ALL the EDI-related themes;
- 0 results on kinesiology, in terms of EDI curriculum and Educators/mentors role;
- 0 results on kinesiology, in terms of EDI/EDI narrative.

#### 3.3.1. University Setting and Sports

##### EDI Recommendation/EDI Needs

There were four findings, in a total of two sources, that discussed EDI recommendation/EDI needs in the context of sport in a university setting. Out of those four findings, none of them discussed a specific EDI group. One of the findings presented the recommendation that, for the future of college recreational sports programs, those programs have a responsibility to address the needs of the changing demographic interests, as well as the diverse students on campus [214]. This source also addressed that, as there is growing diversity on college campuses, the future of college campuses must prioritize EDI [214]. Furthermore, it was recommended that the National Collegiate Athletic Association should have penalties in place for institutions that fail to implement EDI initiatives or for those that do not have any EDI initiatives in place [222]. In addition, recommendations around EDI frameworks and leadership strategies were also presented specifically by addressing that culturally responsive leadership strategies are important for achieving EDI in college sports [222]. 

#### 3.3.2. University Settings and Kinesiology

The recommendation in this one source addressed that the kinesiology program design can use student narratives and experiences to make the shift from neutral documents and pedagogy to ones that expose and work towards dismantling Eurocentricity within the field of study [87].

## 4. Discussion

The objective of this study was to ascertain to what extent (and how) the academic literature focusing on sports, kinesiology, physical education, and physical activity engages with the various EDI phrases and frameworks, as well as which of the marginalized groups covered under EDI are mentioned in the literature covered.

We found only 18 relevant hits with all our search strategies, whereby the EDI frameworks were not at all found. Only ‘sport*’ generated any hits related to EDI phrases; the other three fields did not. The majority of our findings were based on the presence of all the individual EDI terms, but not as phrases; within these sources, the term “sport*” was the most linked to EDI, with much less physical activity or physical education and even less kinesiology. On top, we found a very low to no coverage of marginalized groups normally linked to EDI, namely racialized minorities (12), women (6), LGBTQ2S+ (5), disabled people (2), and Indigenous peoples (0), within the already low coverage of EDI.

Altogether, our findings suggest a huge gap in the academic inquiry and huge opportunities for research on EDI within sports, physical education, physical activity, and kinesiology by themselves, but also in collaborations with many other fields and groups, such as disability studies and other identity group studies, social justice studies, education, media studies, global south focused studies, sustainability studies, socially disadvantaged groups, practitioners, and policy makers. Given that ableism is employed in the academic literature covering sports, physical education, physical activity, and kinesiology, we especially see opportunities for sports, physical education, physical activity, and kinesiology academic efforts to use the ableism lens to enrich the EDI discourses. For the remainder of the section, we discuss the problems of our findings, using as lenses: (a) the academic literature related to physical education, sport, physical activity, and kinesiology, individually covering the terms equity, equality, inclusion, and diversity; (b) the premise of the EDI frameworks and phrases, in general, as well as in the context of disabled people; and (c) ableism experienced by disabled people, but also beyond.

### 4.1. The EDI Policy Frameworks

Efforts performed under the EDI frameworks and EDI policy terms are envisioned to lead to systemic positive change for students, academic staff, and non-academic staff in universities, as a workplace, in general, but also in the research and education reality in universities [1,23,24,25,26,27,34,35,36]. To quote from the Canadian EDI framework DIMENSIONS: Equity, diversity, and inclusion: “Canada invites you to take part in a post-secondary transformation to increase equity, diversity and inclusion (EDI) and help drive deeper cultural change within the research ecosystem” [35] and “The Dimensions program addresses obstacles faced by, but not limited to, women, Indigenous Peoples, persons with disabilities, members of visible minorities/racialized groups, and members of LGBTQ2+ communities” [35].

Given this sweeping mandate for positive systemic and cultural changes EDI actions are to engender, much more should have been found in our searches that link sports, physical education, physical activity, and kinesiology to EDI policy frameworks and phrases with particular emphasis on the EDI targeted groups. However, our findings are not surprising, as the EDI frameworks and phrases are rarely visible in other contexts either, such as disabled people, in general [1]. However, given the very focus of sports, physical education, physical activity, and kinesiology, namely their narratives around the ability of the body and social role of sports, physical education, physical activity, and kinesiology, we suggest they are uniquely situated to contribute and critically analyze EDI discourses, including the ability premises of EDI discourses. Furthermore, disabled students, graduate and undergraduate, are uniquely situated to contribute to this analysis, but there are problems to achieve that goal for disabled students on the undergraduate and graduate levels [1,223,224].

### 4.2. Individual EDI Terms in Sport, Physical Education, Physical Activity, and Kinesiology

In sports, physical education, physical activity, and kinesiology, the individual terms of equity, equality, diversity, and inclusion are discussed, in the context of the EDI groups of gender, race, LGBTQ2S+ [37,38,39,40,41,42,43,44,45,46,47,48,49,50,51,54,55,56,57,58,59,60,61,83,84,85,86,87,88,89,101,102,103,107,108,109,110,111,112,113,122,123,124,125,126], and Indigenous people [62,63,64,65,66,67,115,116,117], including using an intersectionality lens [52,53,90,91,92,93,104,105,106,127,128,129,130]. Individual EDI terms are also discussed in sports, physical education, physical activity, and kinesiology, in the context of disabled people [137,138,139,140,141,142,149], whereby the very meaning of the individual terms, such as diversity and inclusion, are debated [113,151]. All these documents suggest that there could, and should, have been much more coverage of the EDI phrases and frameworks than we found. Our findings are another example of a disconnect between policy terms, frameworks, and existing academic literature. Our findings also suggests that the very conceptual thinking around EDI and sports, physical education, physical activity, and kinesiology might be underdeveloped. Using individual terms, such as equity, equality, diversity, and inclusion, is much easier than using a framework that uses them together (equity, diversity, and inclusion; equality, diversity, and inclusion). Indeed, discussions are ongoing how to fill the individual terms with meaning in the EDI policy frameworks, and more terms are added to these phrases, such as belonging, justice, dignity, accessibility, accountability, and decolonization [2,3,4,5,6,7,8,9,10,11,12,13,14,15,16,17,18,19,20,21,22], suggesting that the initial EDI phrases did not lead to the desired EDI changes anticipated. These ongoing discussions suggest an opening for the academic engagement with the EDI arena in the university setting by sports, physical education, physical activity, and kinesiology. Then the problems and actions flagged as needed in the academic literature covering sport, physical education, physical activity, and kinesiology, as well as the individual EDI terms, are reasons, by themselves, that there could (and should) be more academic engagement of sport, physical education, physical activity, and kinesiology with the EDI phrases and frameworks, in order to generate data on how to make them useful. The existing literature suggests the possibilities of interdisciplinary collaborations on EDI between sports, physical education, physical activity, and kinesiology, as well as other academic fields, where problems of marginalized groups, in the context of sports, physical education, physical activity, and kinesiology, are noted, such as media studies [70,71,72], departments and programs that cover the global south [69], teacher education (including physical education teachers) [113,118,119,120,121,144,145,146,147,149,150,151], and fields covering health, environmental design, and urban design [131,132,133,134,135]. EDI engagement by sports, physical education, physical activity, and kinesiology could be used to engage with many social problems in the community linked to sports, physical education, physical activity, and kinesiology, as well as problems flagged within university settings of sports, physical education, physical activity, and kinesiology, such as group cohesion [73,74], identity formation [75], need for changing curricula [88,96,97], need for diversifying students and faculty [97,98], and need for questioning oppressive discourses and privileges [152].

### 4.3. The Issue of Ableism

Ableism is a term coined by disabled activists and academics to flag the cultural reality of ability-based expectations, judgments, norms, and conflicts [153,154,155,156,157,158,159,160,161,162,163,164,165,166,167,168,169,170,171,172,173,174,175,176]. Ableism is also used as a conceptual framework, to call out ability-based discriminations against disabled people within the kinesiology, sports, physical education, and physical education literature [91,149,151,183,184,185,186,187,188,189,190,191,192,193,194]. Ableism is seen as one reason for the invisibility of disabled people and their problems in many subject topics and degrees [196], and it is argued that it is critical to understand “ableism and how ableistic ideology informs sporting theory, sport science and sport management” [197] (p. 5), and a “different ability curriculum, which destabilises notions of normativity and challenges the status quo regarding ability” is needed [194] (p. 518). As such, the problems indicated in sports, physical education, physical activity, and kinesiology, under the concept of ableism, suggest the need for critical evaluation of EDI frameworks and policies developed under EDI phrases that are applied to disabled people, which sports, physical education, physical activity, and kinesiology are well situated to provide. However, ableism is more useful.

#### Ableism beyond Disabled People

The cultural reality of ableism is intersectional [178,179,180,181]. The intersectionality of experiencing different forms of isms, including ableism and problematic ability, linked prejudices, perceptions, microaggression, discrimination, language, and attitudes toward disabled people and others are highlighted in the education of students and curricula in sports, physical education, physical activity, and kinesiology [183,184,185,186,191,193]. It is argued that experiences and discourses around sport often favor and internalize ableism [183,187,190]. However, the very cultural reality of ableism goes beyond being part of a list of isms. Ableism is used to enable other negative isms, such as sexism, racism, classism, and ageism, that have, at their core, ability judgments [153,154,182]. Masculinity and supercrip identities, mentioned in [183], are not just internalized identities similar to the ableism identity mentioned in [183]; abilities are used to justify masculinity by itself, indeed masculinity is seen as an essential ability and a lack of such is used to question certain social groups [182]. Ableism is flagged as a useful analytical perspective in sports pedagogy to reflect on its discriminatory and exclusionary potential [149]; there are there are competing ability-related narratives [149]. Ableism could be used by sports, physical education, physical activity, and kinesiology as a conceptual framework to engage with all EDI covered groups, because all EDI groups are judged based on abilities (body-derived, culture-derived). We think sports, physical education, physical activity, and kinesiology are uniquely positioned to engage with, and enrich, the discussions around the cultural intersectional reality of ability-based expectations, judgments, norms, and conflicts, in conjunction with EDI frameworks and policy work. Sports, physical education, physical activity, and kinesiology can make use of many ability concepts, such as internalized ableism [168], ability privilege [225], ability security (one is able to live a decent life with whatever set of abilities one has), ability identity security (to be able to be at ease with ones abilities), and ability inequity, an unjust or unfair (a) “distribution of access to and protection from abilities generated through human interventions” or (b) “judgment of abilities intrinsic to biological structures such as the human body” [156,177] to enrich the EDI discussions. Sports, physical education, physical activity, and kinesiology can enrich the ability-based EDI discussions, as well, by engaging with the area of human body ability enhancements obtained through, for example, drugs, genetic and cybernetic interventions, the linked consequences of ability creeps (expecting more and more abilities of the human body; physical, mental and cognitive), and ability obsolescence [226].

### 4.4. Limitations

The search was limited to two academic databases and English language literature. As such, the findings are not to be generalized to the whole academic literature, non-academic literature, or non-English literature. These findings, however, allow conclusions to be made, within the parameters of the searches.

## 5. Conclusions and Future Research

The low hits we obtained in our scoping review suggests a gap in academic inquiry around EDI and sports, physical education, physical activity, and kinesiology. Many research projects could evaluate the existing EDI frameworks and EDI phrases, through the lens of sports, physical education, physical activity, and kinesiology. One could answer the following research questions: What do the EDI phrases mean for sports, physical education, physical activity, and kinesiology? Which phrases are the most suitable for sports, physical education, physical activity, and kinesiology? Do words have to be added to the phrases? Why has the academic literature in sports, physical education, physical activity, and kinesiology not engaged with the phrases and frameworks yet? What are the societal consequences of sports, physical education, physical activity, and kinesiology not generating academic data and engaging with the EDI frameworks and phrases? Answers to these questions can be obtained conceptually for sports, physical education, physical activity, and kinesiology, with surveys and interviews covering EDI-deserving groups on the level of students, academic staff, and non-academic staff. This research can engage with workplace climate, education, and research realities. For example, in a 2019 Statistics Canada survey, it is stated that 35% of disabled university professors, instructors, teachers, or researchers “experienced unfair treatment or discrimination in the past 12 months”, and 47% saw themselves “subjected to at least one type of harassment in the past 12 months” [227]. Within this survey, the numbers for disabled university professors, instructors, teachers, or researchers are the highest of all groups listed [227]. The respective numbers for “no self-reported disability” were 15.4% and 26.0%; “female gender” were 23.0% and 34.0%; “visible minorities” were 23.0% and 28.0%; and “indigenous identity” were 30.0% and 37.0%” [1] (p. 5). Such a reality must impact the implementation of positive systemic and cultural EDI changes in universities. One could generate numbers specific for sports, physical education, physical activity, and kinesiology activities at universities. We also suggest that sports, physical education, physical activity, and kinesiology activities at universities are uniquely situated to perform conceptual and empirical work on linking ableism to EDI in their areas and beyond. We suggest that sports, physical education, physical activity, and kinesiology all focus on the body, and the body is the primary source of ability judgments. These judgments are then used to enable some groups/individuals over others or disable some groups/individuals. We see sports, physical education, physical activity, and kinesiology to be uniquely situated to engage with the linkage of body ability judgments and non-body linked abilities, such as competitiveness, productivity, or the ability to have a good life [228]. We also see that sports, physical education, physical activity, and kinesiology uniquely positioned to generate and run ability-based surveys that could enrich EDI discourses.

## Figures and Tables

**Table 1:** Search strategies.

Strategy	Sources Used	First Search
Strategy 1	Scopus/EBSCO-Host	ABS (“Athena SWAN” OR “See change with STEMM Equity Achievement” OR “Dimensions: equity, diversity and inclusion” OR “Science in Australia Gender Equity” OR “NSF ADVANCE” OR “equity, diversity and inclusion” OR “equality, diversity and inclusion” OR “diversity, equity and inclusion” OR “diversity, equality and inclusion”) AND ABS (“Kinesiology” OR “physical education” OR “physical activit*” OR “sport*”)
Strategy 2	Scopus/EBSCO-Host	ABS (“Belonging, Dignity, and Justice” OR “Diversity, Equity, Inclusion and Belonging” OR “diversity, Dignity, and Inclusion” OR “Equity, Diversity, Inclusion, and Accessibility” OR “Justice, Equity, Diversity, and Inclusion” OR “Inclusion, Diversity, Equity and Accessibility” OR “Inclusion, Diversity, Equity and Accountability” OR “Equity, Diversity, Inclusion, and Decolonization”) AND ABS (“kinesiology” OR “physical education” OR “physical activit*” OR “sport*”)
Strategy 3a	Scopus/EBSCO-Host	ABS (“equity” AND “diversity” AND “inclusion”) AND ABS (“kinesiology” OR “physical education” OR “physical activit*” OR “sport*”)
Strategy 3b	Scopus/EBSCO-Host	ABS (“equality” AND “diversity” AND “inclusion”) AND ABS (“kinesiology” OR “physical education” OR “physical activit*” OR “sport*”)

**Table 2:** Frequency of themes related to EDI and Physical education in an academic/educational setting.

Area of Coverage	Degree of Coverage	EDI-Related Theme	EDI-Related Equity Deserving Groups Mentioned	Result
Academic/educational Setting	Physical Education	EDI recommendation/EDI needs	Women	0
			Disabled People	1
			LGBTQ2S+	0
			Racialized Minorities	3
			Indigenous Peoples	0
			No group	8
		EDI Curriculum and Educators and Mentors role in EDI	Women	0
			Disabled People	0
			LGBTQ2S+	0
			Racialized Minorities	1
			Indigenous Peoples	0
			No group	8
		EDI Literacy/EDI Narrative	Women	0
			Disabled People	0
			LGBTQ2S+	0
			Racialized Minorities	0
			Indigenous Peoples	0
			No group	2

**Table 3:** Frequency of themes related to EDI and sports in an academic/educational setting.

Area of Coverage	Degree of Coverage	EDI-Related Theme	EDI-Related Equity Deserving Groups Mentioned	Result
Academic/educational Setting	Sports	EDI recommendation/EDI needs	Women	0
			Disabled People	0
			LGBTQ2S+	0
			Racialized Minorities	0
			Indigenous Peoples	0
			No group	3
		EDI literacy/EDI narrative	Women	0
			Disabled People	0
			LGBTQ2S+	0
			Racialized Minorities	0
			Indigenous Peoples	0
			No group	1
		EDI study result	Women	2
			Disabled People	1
			LGBTQ2S+	1
			Racialized Minorities	3
			Indigenous Peoples	0
			No groups	3

**Table 4:** Frequency of themes related to EDI and physical activity in a non-academic setting.

Area of Coverage	Degree of Coverage	EDI-Related Theme	EDI-Related Equity Deserving Groups Mentioned	Result
Non-academic setting	Physical activity	EDI recommendation/EDI needs	Women	0
			Disabled People	0
			LGBTQ2S+	0
			Racialized Minorities	0
			Indigenous Peoples	0
			No group	1

**Table 5:** Frequency of themes related to EDI and sports in a non-academic setting.

Area of Coverage	Degree of Coverage	EDI-Related Theme	EDI-Related Equity Deserving Groups Mentioned	Result
Non-academic setting	Sports	EDI recommendation/EDI needs	Women	1
			Disabled people	0
			LGBTQ2S+	0
			Racialized Minorities	3
			Indigenous Peoples	0
			No group	2
		EDI study result	Women	3
			Disabled People	0
			LGBTQ2S+	4
			Racialized Minorities	2
			Indigenous Peoples	0
			No groups	0

**Table 6:** Frequency of themes related to EDI and sports in a university setting.

Area of Coverage	Degree of Coverage	EDI-Related Theme	EDI-Related Equity Deserving Groups Mentioned	Result
University Setting	Sports	EDI recommendation/EDI needs	Women	0
			Disabled people	0
			LGBTQ2S+	0
			Racialized Minorities	0
			Indigenous Peoples	0
			No group	4

**Table 7:** Frequency of themes related to EDI and kinesiology in a university setting.

Area of Coverage	Degree of Coverage	EDI-Related Theme	EDI-Related Equity Deserving Groups Mentioned	Result
University Settings	Kinesiology	EDI recommendation/EDI needs	Women	0
			Disabled People	0
			LGBTQ2S+	0
			Racialized Minorities	0
			Indigenous Peoples	0
			No groups	1

## Data Availability

Data sharing is not applicable to this article.
